# Anomalous Left Circumflex Artery With ST Depressions and T-wave Inversions in a Healthy Athlete: A Case Report

**DOI:** 10.7759/cureus.78777

**Published:** 2025-02-09

**Authors:** Anne V Sauber, Yash Patil, Allan Stahl, Erek M Humm, Sarah Legrand, Harish Venkat

**Affiliations:** 1 Cardiology, Touro University Nevada, Las Vegas, USA; 2 Cardiology, Prime Cardiology Nevada, Las Vegas, USA; 3 Internal Medicine, Touro University Nevada, Las Vegas, USA

**Keywords:** anomalous left circumflex artery, coronary artery angiography, coronary vessel anomaly, ct coronary angiography, right coronary artery (rca), st changes, st-segment abnormalities, st-segment depression, t wave, t-wave inversion

## Abstract

An anomalous left circumflex artery is a congenital anatomic variant of typical coronary circulation that can potentially contribute to cardiac ischemia or altered blood flow. These variants can cause changes to be seen on electrocardiograms, particularly to the T wave and ST segments, including depressions, inversions, or elevations. We describe the case of a healthy 35-year-old man with previously undiagnosed anomalous origin of the left circumflex artery from the right coronary artery ostium with evidence of ST-segment changes and T-wave inversions on electrocardiogram during exercise stress testing, despite the lack of evidence of atherosclerosis and the patient being in good cardiac health otherwise. Anomalous left circumflex arteries can present with altered hemodynamics in some patients, particularly during strenuous exercise, as this arterial change disrupts the typical cardiac blood flow. Repeat stress testing done with a treadmill myocardial perfusion study prior to the patient's participation in a marathon showed no persistence of the ST changes. Given the reassuring findings of the lack of ST changes and the patient having no symptoms of ischemia, such as chest pain or shortness of breath, the patient was advised to continue his current activity level while diligently monitoring for symptoms of myocardial ischemia. This case shows that even though this anomaly can be asymptomatic, ST changes can potentially occur due to altered hemodynamics in the coronary circulation that could require additional monitoring.

## Introduction

Coronary artery anomalies are defined as abnormalities in the artery's origin, course, termination, or morphology [1]. Although often clinically silent, an anomaly of the coronary arteries can be associated with an increased risk of sudden cardiac death, myocardial ischemia, hypertension, and myocardial infarction, especially during bouts of strenuous exercise [1]. Most often, diagnosis of aberrant coronary artery is an incidental finding on non-invasive and invasive cardiac imaging for follow-up on a heart murmur noted on a physical exam or an abnormal EEG, and treatment can range from minimizing strenuous exercise to percutaneous intervention and revascularization [2].

The most common variation of cardiac circulation applies to the concept of right and left circulatory dominance. Most of the population (70-80%) is considered "right heart dominant," indicating that the posterior descending artery (PDA) originates from the right coronary artery; however, there is a small percentage (5-10%) of the population that is "left heart dominant" with the PDA originating from the left coronary artery [3]. Approximately 10-20% of the population is "codominant," with the PDA getting dual blood supply by both the right and left coronary arteries [3]. The dominance in circulation becomes clinically significant due to the PDA supplying the atrioventricular (AV) node, which can become stenosed based on circulatory dominance, leading to arrhythmias and possibly cardiac death.

Furthermore, five coronary artery anomalies are seen in approximately 0.2-1.2% of the population, with the predominant anomaly being the left circumflex artery (LCx) branching from a location other than the left coronary artery [4]. This anomalous origin is often from the right coronary artery and typically passes underneath the great vessels. It is typically a benign condition, but in rare cases, it can be associated with dyspnea and chest pain on exertion or arrhythmias with strenuous exercise. In a study examining 27 cases of coronary artery anomalies conducted by Frescura et al. [4], the aberrant origin of the LCx artery was only associated with one clinical case of significant obstructive multivessel atherosclerosis, implying that an aberrant origin of the LCx artery is a relatively benign condition with rare comorbidities [5]. Rarely, ST-segment elevations can be present with myocardial ischemia when an aberrant LCx is present [6]. Clinically, the ST segment is the part of an electrocardiogram (ECG) between the S and T points where the myocardium is contracting to pump out blood from the ventricles. An ST depression can be measured when the J point, the connection between the QRS complex and ST segment, is seen below the baseline and can represent conditions such as myocardial ischemia, medication toxicity, hypokalemia, or be unrelated to an emergent condition. In addition, T waves typically indicate ventricular repolarization, and abnormalities such as T-wave inversions can indicate conditions such as myocardial ischemia, bundle branch blocks, hypertrophic cardiomyopathy, or a normal variant [7]. This case involves a 35-year-old man diagnosed with an anomalous LCx on coronary angiogram with ST-segment depressions and T-wave inversions on exercise stress testing.

## Case presentation

**Figure 1:**
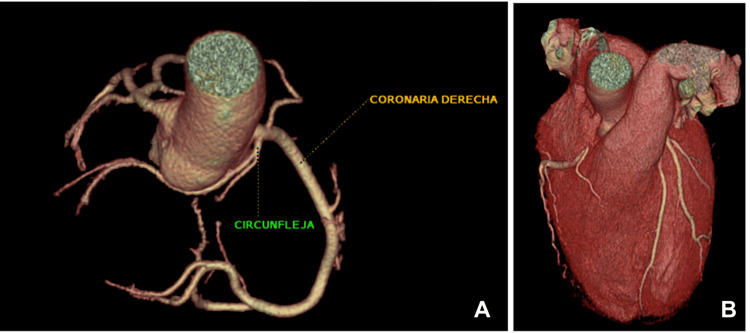
Computed tomography angiography of the heart coronaries (A) and myocardium (B) Coronaria derecha: right coronary artery; circunfleja: circumflex Original image by the authors

A 35-year-old Ukrainian man presented to Prime Cardiology Henderson on September 2024 with a history of anomalous origin of the LCx next to the right coronary artery noted on a computed tomography (CT) angiogram done in Columbia (Figure 1). The LCx was noted to arise from the right coronary artery ostium and follow a retroaortic course toward its usual position in the left atrioventricular groove. The patient reported that during his teenage years, he was informed of having mitral valve prolapse and that it could potentially resolve with age. Currently, no murmur due to mitral valve prolapse was heard on auscultation. He has varicose veins and no other diagnosed medical conditions, does not drink alcohol or smoke tobacco, and maintains a healthy diet. The family history is remarkable for his mother, who died at the age of 62 from a ruptured abdominal aorta, having suffered from systemic lupus erythematosus and varicose veins. His father has noted having an arrhythmia, but no further workup has been conducted at this time.

The patient has no history of myocardial infarction and frequently runs half-marathons and marathons, having run 15 half-marathons in the last year. He has an extensive workout routine consisting mainly of cardiothoracic exercise and weight lifting, which he completes every week, Monday through Sunday. He reports running distances totaling 100 km per week at varying intensities mixed in with calisthenics during the preparation. He initially sought a cardiology workup due to his preparation for a marathon and concerns regarding possible cardiac risks associated with the strenuous exercise.

**Figure 2:**
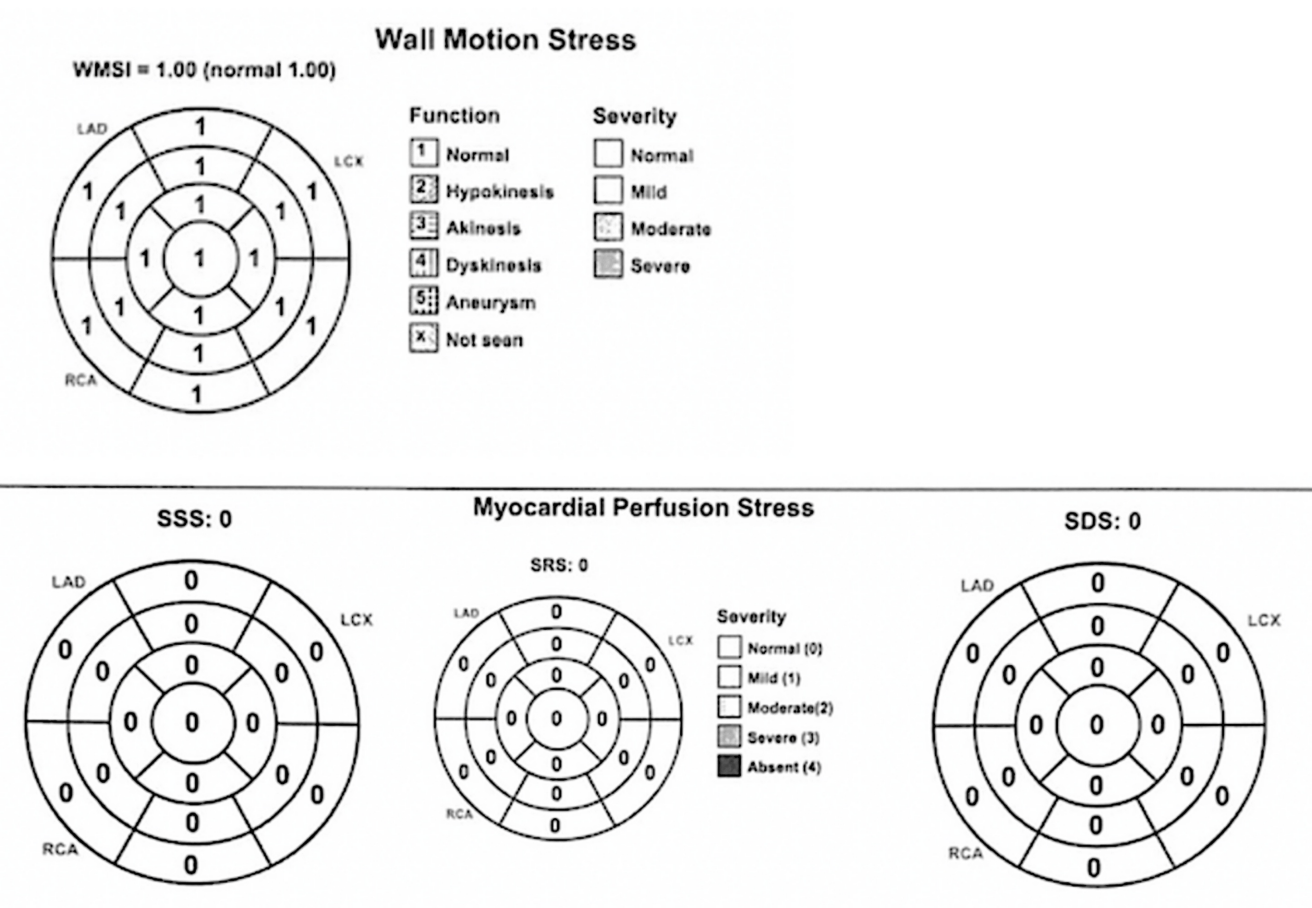
Myocardial perfusion scan WMSI: wall motion score index; LAD: left anterior descending artery; LCx: left circumflex artery; RCA: right coronary artery; SSS: summed stress score; SRS: summed rest score; SDS: summed difference score Original image by the authors

On physical examination, the patient is 77 inches tall and well-appearing; he was normotensive and in no acute distress; the heart exam showed a regular rate with no murmurs or gallops, and the lungs were clear with no wheezes, rhonchi, or rales. Peripheral pulses were assessed at a grade of 2+ consistently, with capillary refill time recorded at less than two seconds. There was no peripheral edema or clubbed digits. The skin was unremarkable with no mottling, discoloration, or lesions seen. He was recommended to have a myocardial perfusion scan (Figure 2) to evaluate for any ischemic changes due to the coronary anomaly, which was conducted in September 2024. The single-photon emission computed tomography (SPECT) Regadenoson with Treadmill Myocardial Perfusion Study Report showed no evidence of ischemia with normal myocardial perfusion and a stress left ventricular ejection fraction of 73%.

**Figure 3:**
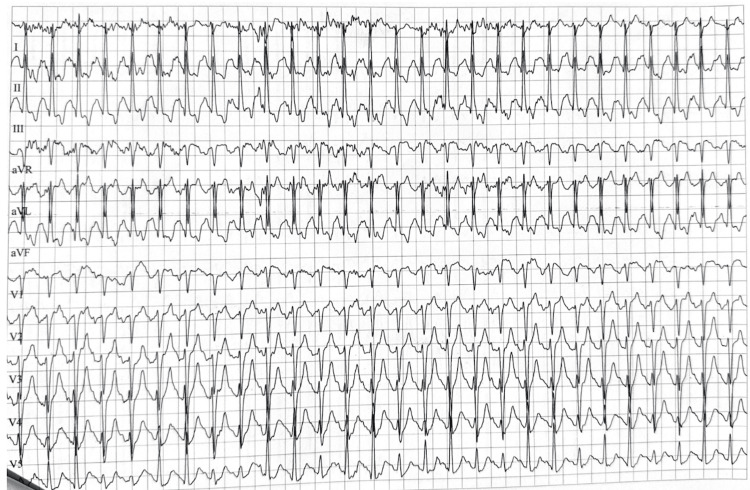
Stress test electrocardiogram

On exercise treadmill stress testing with ECG done in Columbia, the patient was noted to have significant ST depressions and T-wave inversions at the inferior wall (leads II, III, and aVF) at peak stress (Figure 3), consistent with New York Heart Association (NYHA) class I [8], which was used as a measurement of functional capacity, despite the patient having no history of chest pain even at peak exercise. ST depression was a maximum of 425 microvolts, persisting through peak exercise and resolving at rest. This was considered to be an abnormal stress test, given the changes discussed and the lack of symptoms from the patient. The patient also had a lipid panel performed in August 2024, which was unremarkable except for a low-density lipoprotein (LDL) cholesterol of 109 mg/dL (Table 1). The lipid panel also included high-density lipoprotein (HDL) and cholesterol (chol) to HDC cholesterol (HDLC) ratio.

**Table 1:** Standard lipid panel HDL: high-density lipoprotein; LDL: low-density lipoprotein; Chol: cholesterol; HDLC: high-density lipoprotein cholesterol; mg: milligrams; dL: deciliters; calc: calculated

Parameters	Result	Reference Range	Remarks
Total cholesterol	163 mg/dL	<200	-
HDL cholesterol	41 mg/dL	> OR = 40	-
Triglycerides	44 mg/dL	< 150	-
LDL cholesterol	109 mg/dL (calc)	< 100 (for primary prevention)	High
Chol/HDLC ratio	4.0 (calc)	< 5.0	-
Non-HDL cholesterol	122 mg/dL (calc)	< 130	-

The patient exercised according to the Bruce protocol for 13:29 minutes, in the excellent range with metabolic equivalents (METs) of 17.20 [9] with a resting heart rate of 69 beats per minute (bpm), reaching a maximum heart rate of 173 bpm. The resting blood pressure was 100/70 mmHg, with a maximum of 160/40 mmHg reached during testing. A high pulse pressure in this instance may be indicative of decreased vascular compliance but is less concerning in the context of a normal resting blood pressure and lack of persistent ischemic changes on further follow-up. There was no arrhythmia during this test. This showed the anomalous origin of the circumflex artery from the right coronary ostium and patent coronary arteries without atherosclerotic disease, although it was electrically positive for coronary insufficiency.

The patient came to follow up on the previously mentioned myocardial perfusion scan at Prime Cardiology Siena in October 2024. The physical examination remained consistent with the prior assessment. The patient remains asymptomatic with an unremarkable follow-up ECG showing no persistence of the ST and T-wave changes and was informed that he could continue his exercise regimen and to follow up in one year. He was additionally instructed to closely monitor himself for concerning signs and symptoms during exercise, such as angina, dyspnea, dizziness, light-headedness, and palpitations.

## Discussion

This case highlights the anomalous origin of the LCx from the right coronary artery ostium in a healthy, active 35-year-old man with a variant right dominant circulation. This is a relatively common congenital anatomical variant that is typically benign [10], although it can potentially be associated with increased cardiovascular risks during periods of strenuous exercise. Potential mechanisms include altered hemodynamics, vessel trajectory leading to external compressions, or turbulent blood flow, which could compromise myocardial perfusion. In particular, high-output states during intense exercise could accentuate these risks due to dynamic compression or transient vessel deformation.

In this case, the patient's asymptomatic ST-segment depressions and T-wave inversions on the initial exercise treadmill stress test taken in South America, which lasted 13:29 minutes, raised concerns about possible subclinical ischemic changes during high-intensity exertion. These transient ischemic changes, despite the absence of atherosclerosis and the patient's high functional capacity, may stem from mechanical factors related to the anomalous course of the LCx. It is possible there could have been a compression of the anomalous vessel during systole or by adjacent structures that could have led to compromised coronary flow during the high-intensity exertion. In addition, altered blood flow in this anomalous LCx, often known as "vestigial" due to its small area of myocardial perfusion and positioning, may predispose this patient to localized myocardial ischemia even when overall myocardial oxygen demand remains low.

However, the initial stress test findings were not reproduced on a follow-up nuclear stress test, which showed no ischemic changes. These findings are reassuring since nuclear stress tests provide more advanced and detailed information. Nuclear stress tests utilize a radioactive tracer that produces an image of the blood flow in the heart that allows us to determine how well blood is being perfused through different regions of the heart. A treadmill stress test measures the electrical activity of the heart during exercise through an EKG and provides information on changes in heart rhythm. Therefore, the discrepancy between the two stress tests may be attributable to the situational nature of the ischemic changes. Some possible factors to consider are hydration status, neurohormonal shifts, human error, and testing conditions. This case corresponds with prior results by Frescura et al., which demonstrated that although coronary artery anomalies are typically benign [4], rare cases may show ischemic or obstructive pathologies in the presence of strenuous physical activity. Overall, the absence of ST-segment depressions and T-wave inversions on the nuclear stress tests supports that the initial findings were likely due to situational factors as opposed to persistent, structural abnormalities.

Further evaluation of this patient revealed a normal lipid panel, with LDL cholesterol levels within manageable limits. The lipid panel supports the absence of atherosclerotic disease, which further reduces the patient's cardiovascular risk profile in addition to the normal nuclear stress test.

Given the asymptomatic patient's promising nuclear stress test and lipid profile results, it appears safe for him to maintain his present level of physical activity and continue participating in marathons. However, he was advised to monitor for any new symptoms, such as chest pain, palpitations, or dyspnea, and to have follow-up evaluations to ensure continued cardiovascular health.

## Conclusions

This case study describes a 35-year-old active man with an anomalous LCx originating from the right coronary artery, a common but typically benign congenital variant. Despite his lack of atherosclerosis and good physical fitness, he showed ST-segment depressions and T-wave inversions during an exercise stress test, raising concerns for myocardial ischemia during high-intensity exertion. Several factors that may need to be considered include neurohormonal shifts, such as those due to stress or fatigue, and hydration status, as dehydration can significantly impact performance and physiological responses. The relationship between the abnormal LCx and potential ischemia does not show a definitive correlation in the presented data; more research on this topic needs to be conducted to identify a strong correlation, given that this patient showed laboratory evidence of ischemia without having symptoms. The absence of these symptoms, such as chest pain or shortness of breath, alongside these findings of a nuclear stress test with no ischemic changes, suggesting temporary factors may have influenced the initial test results, demonstrates an atypical presentation inconsistent with known symptoms accompanying myocardial ischemia. This information supports the decision to allow the patient to continue his exercise routine, including marathons, while advising him to monitor for new symptoms such as chest pain or palpitations and to have follow-up evaluations to maintain cardiovascular health.
